# Networks and the Epidemiology of Infectious Disease

**DOI:** 10.1155/2011/284909

**Published:** 2011-03-16

**Authors:** Leon Danon, Ashley P. Ford, Thomas House, Chris P. Jewell, Matt J. Keeling, Gareth O. Roberts, Joshua V. Ross, Matthew C. Vernon

**Affiliations:** ^1^School of Life Sciences, University of Warwick, Coventry CV4 7AL, UK; ^2^Department of Statistics, University of Warwick, Coventry CV4 7AL, UK; ^3^Warwick Mathematics Institute, University of Warwick, Coventry CV4 7AL, UK; ^4^School of Mathematical Sciences, University of Adelaide, SA 5005, Australia

## Abstract

The science of networks has revolutionised research into the dynamics of interacting elements. It could be argued that epidemiology in particular has embraced the potential of network theory more than any other discipline. Here we review the growing body of research concerning the spread of infectious diseases on networks, focusing on the interplay between network theory and epidemiology. The review is split into four main sections, which examine: the types of network relevant to epidemiology; the multitude of ways these networks can be characterised; the statistical methods that can be applied to infer the epidemiological parameters on a realised network; and finally simulation and analytical methods to determine epidemic dynamics on a given network. Given the breadth of areas covered and the ever-expanding number of publications, a comprehensive review of all work is impossible. Instead, we provide a personalised overview into the areas of network epidemiology that have seen the greatest progress in recent years or have the greatest potential to provide novel insights. As such, considerable importance is placed on analytical approaches and statistical methods which are both rapidly expanding fields. Throughout this review we restrict our attention to epidemiological issues.

## 1. Introduction

The science of networks has revolutionised research into the dynamics of interacting elements. The associated techniques have had a huge impact in a range of fields, from computer science to neurology, from social science to statistical physics. However, it could be argued that epidemiology has embraced the potential of network theory more than any other discipline. There is an extremely close relationship between epidemiology and network theory that dates back to the mid-1980s [1, 2]. This is because the connections between individuals (or groups of individuals) that allow an infectious disease to propagate naturally define a network, while the network that is generated provides insights into the epidemiological dynamics. In particular, an understanding of the structure of the transmission network allows us to improve predictions of the likely distribution of infection and the early growth of infection (following invasion), as well as allowing the simulation of the full dynamics. However the interplay between networks and epidemiology goes further; because the network defines potential transmission routes, knowledge of its structure can be used as part of disease control. For example, contact tracing aims to identify likely transmission network connections from known infected cases and hence treat or contain their contacts thereby reducing the spread of infection. Contact tracing is a highly effective public health measure as it uses the underlying transmission dynamics to target control efforts and does not rely on a detailed understanding of the etiology of the infection. It is clear, therefore, that the study of networks and how they relate to the propagation of infectious diseases is a vital tool to understanding disease spread and, therefore, informing disease control.

Here, we review the growing body of research concerning the spread of infectious diseases on networks, focusing on the interplay between network theory and epidemiology. The paper is split into four main sections which examine the types of network relevant to epidemiology, the multitude of ways these networks can be characterised, the statistical methods that can be applied to either infer the likely network structure or the epidemiological parameters on a realised network, and finally simulation and analytical methods to determine epidemic dynamics on a given network. Given the breadth of areas covered and the ever-expanding number of publications (over seven thousand papers have been published concerning infectious diseases and networks) a comprehensive review of all work is impossible. Instead, we provide a personalised overview into the areas of network epidemiology that have seen the greatest progress in recent years or have the greatest potential to provide novel insights. As such considerable importance is placed on analytical approaches and statistical methods which are both rapidly expanding fields. We note that a range of other network-based processes (such as the spread of ideas or panic) can be modelled in a similar manner to the spread of infection; however, in these contexts, the transmission process is far less clear; therefore, throughout this paper, we restrict our attention to epidemiological issues.

## 2. Networks, Data, and Simulations

There are a wide number of network structures and types that have been utilised when considering the spread of infectious diseases. Here, we consider the most common forms and explain their uses and limitations. Later, we review the implications of these structures for the spread and control of infectious diseases.

### 2.1. The Ideal Network

We start our examination of network forms by considering the ideal network that would allow us to completely describe the spread of any infectious pathogen. Such a network would be derived from an omniscient knowledge of individual behaviour. We define *G*_*i*,*j*_(*t*) to be a time-varying, real, and high-dimensional variable that informs about the strength of all potential transmission routes from individual *i* to individual *j* at time *t*. Any particular infectious disease can then be represented as a function (*f*_pathogen_) translating this high-dimensional variable into an instantaneous probabilistic transmission rate (a single real variable). In this ideal, *G* subsumes all possible transmission networks, from sexual relations to close physical contact, face-to-face conversations, or brief encounters, and quantifies the time-varying strength of this contact. The disease function then picks out (and combines) those elements of *G* that are relevant for transmission of this pathogen, delivering a new (single-valued) time-varying infection-specific matrix (*T*_*i*,*j*_(*t*) = *f*_pathogen_(*G*_*i*,*j*_(*t*))). This infection-specific matrix then allows us to define the stochastic dynamics of the infection process for a given pathogen. (For even greater generality, we may want to let the pathogen-specific function *f* also depend on the time since an individual was infected, such that time-varying infectivity or even time-varying transmission routes can be accommodated.)

Obviously, the reality of transmission networks is far from this ideal. Information on the potential transmission routes within a population tends to be limited in a number of aspects. Firstly, it is rare to have information on the entire population; most networks rely on obtaining personal information on participants, and therefore participation is often limited. Secondly, information is generally only recorded on a single transmission route (e.g., face-to-face conversation or sexual partnership) and often this is merely recorded as the presence or absence of a contact rather than attempting to quantify the strength or frequency of the interaction. Finally, data on contact networks are rarely dynamic; what is generally recorded is whether a contact was present during a particular period with little consideration given to how this pattern may change over time. In the light of these departures from the ideal, it is important to consider the specifics of different networks that have been recorded or generated and understand their structure, uses, and limitations.

### 2.2. Realised Encounter Networks

**Figure 1:**
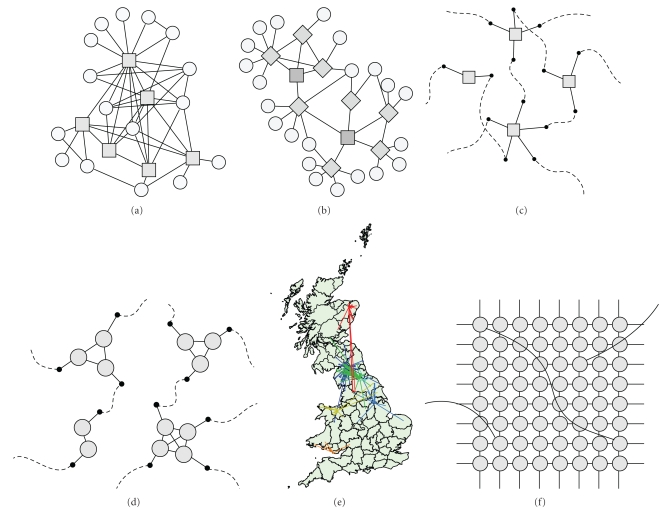
Examples of networks used in epidemiology. (a) Contacts between 22 intravenous drug users, as recorded in [3]; squares refer to primary contacts. Given that the identity of contacts is known, they can be interlinked. (b) Caricature of a snowball sampling algorithm, squares are primary contacts, diamonds are secondary, and circles are tertiary contacts. Given that the identity of contacts is known they can be linked. (c) Example of a configuration model network. Each individual has a prescribed degree distribution, which gives rise to “half-links” that are connected at random. (d) A household configuration network, consisting of completely interconnected households (cliques) with each individual also having one random link to another household. (e) Map showing Great Britain, together with the movements of cattle from six farms (each represented in a separate colour). Notice the heterogeneity between farms and the generally localised nature of movements. (f) Example of a small-world model based on a 2D lattice with nearest neighbor connections. The small-world property is given by the presence of rare random links that can connect distant parts of the network.

One of the few examples of where many of the potential transmission routes within a population have been documented comes from the spread of sexually transmitted infections (STIs). In contrast with airborne infections, STIs have very obvious transmission routes—sex acts (or sharing needles during intravenous drug use)—and as such these potential transmission routes should be easily remembered (Figure 1(a)). Generally the methodology replicates that adopted during contact tracing, getting an individual to name all their sexual partners over a given period, these partners are then traced and asked for their partners, and the process is repeated—this is known as * snowball sampling* [4] (Figure 1(b)). A related methodology is *respondent*-*driven sampling*, where individuals are paid both for their participation and the participation of their contacts while protecting each individual's anonymity [5]. This approach, while suitable for hidden and hard to reach populations, has a number of limitations, both practical and theoretical: recruiting people into the study, getting them to disclose such highly personal information, imperfect recall from participants, the inability to find all partners, and the clustering of contacts. In addition, there is the theoretical issue that this algorithm will only find a single connected component within the population, and it is quite likely that multiple disjoint networks exist [6]. 

Despite these problems, and motivated by the desire to better understand the spread of HIV and other STIs, several pioneering studies were performed. Probably the earliest is discussed by Klovdahl [1] and utilises data collected by the Center for Disease Control from 19 patients in California suffering from AIDS, leading to a network of 40 individuals. Other larger-scale studies have been performed in Winnipeg, Manitoba, Canada [7] and Colorado Springs, Colorado, USA [8]. In both of these studies, participants were tested for STIs, and the distribution of infection compared to the underlying network structure. Work done on both of these networks has generally focused on network properties and the degree to which these can explain the observed cases; no attempt was made to use these networks predictively in simulations. In addition, in the Colorado Springs study, tracing was generally only performed for a single iteration although many initial participants in high-risk groups were enrolled, while in the Manitoba study, tracing was performed as part of the routine information gathered by public health nurses. Therefore, while both provide a vast amount of information on sexual contacts, it is not clear if the results are truly a comprehensive picture of the network and sampling biases may corrupt the resulting network [9]. In addition, compared to the ideal network, these sexual contact networks lack any form of temporal information; instead, they provide an integration of the network over a fixed time period and generally lack information on the potential strength of a contact between individuals. Despite these difficulties, they continue to provide an invaluable source of information on human sexual networks and the potential transmission routes of STIs. In particular, they point to the extreme levels of heterogeneity in the number of sexual contacts over a given period—and the variance in the number of contacts has been shown to play a significant role in early transmission dynamics [10].

One of the few early examples of the simulation of disease transmission on an observed network comes from a study of a small network of 22 injection drug users and their sexual partners [3] (Figure 1(a)). In this work, the risk of transmission between two individuals in the network was imputed based on the frequency and types of risk behaviour connecting those two individuals. HIV transmission was modelled using a monthly time step and single index case, and simulations were run for varying lengths of (simulated) time. This enabled a node's position in the network (as characterised by a variety of measures) to be compared with how frequently it was infected during simulations, and how many other nodes it was typically responsible for infecting.

A different approach to gathering social network and behavioural data was initiated by the Human Dynamics group at MIT and illustrates how modern technology can assist in the process of determining transmission networks. One of the first approaches was to take advantage of the fact that most people carry mobile phones [11]. In 2004, 100 Nokia 6600 smart-phones preinstalled with software were given to MIT students to use over the course of the 2004-2005 academic year. Amongst other things, data were collected using Bluetooth to sense other mobile phones in the vicinity. These data gave a highly detailed account of individuals behaviour and contact patterns. However, a limitation of this work was that Bluetooth has a range of up to 25 meters, and as such networks inferred from these data may not be epidemiological meaningful.

A more recent study into the encounters between wild Tasmanian devils in the Narawntapu National Park in northern Tasmania utilised a similar technological approach [12]. In this work, 46 Tasmanian devils were fitted with proximity loggers that could detect and record the presence of other loggers within a 30 cm range. As such, these loggers were able to provide detailed temporal information on the potential interaction between these 46 animals. This study was initiated to understand the spread of Tasmanian devil facial tumour disease, which causes usually fatal tumours that can be transmitted between devils if they fight and bite each other. Although only 27 loggers with complete data were recovered, and although the methodology only recorded interaction between the 46 devils in the study, the results were highly informative (generating a network that was far from random, heterogeneous, and of detailed temporal resolution). Analyses based on the structure of this network suggested that targeted measures, that focus on the most highly connected ages or sex, were unlikely to curtail the spread of this infection. Of perhaps greater relevance is the potential this method illustrates for determining the contact networks of other species (including humans)—the only limitation being the deployment of a suitable number of proximity loggers.

### 2.3. Inferred Encounter Networks

Given the huge logistical difficulties of capturing the full network of interactions between individuals within a population, a variety of methods have been developed to generate synthetic networks from known attributes. Generally, such methods fall into two classes: those that utilise egocentric information and those that attempt to simulate the behaviour of individuals.

Egocentric data generally consists of information on a number of individuals (the egos) and their contacts (the alters). As such the information gathered is very similar to that collected in the sexual contact network studies in Manitoba and Colorado Springs, but with only the initial step of the snowball sampling was performed; the difference is that for the majority of egocentric data the identity of partners (alters) is unknown and therefore connections between egos cannot be inferred (Figure 1(c)). The data, therefore exists as multiple independent “stars” linking the egos to the alters, which in itself provides valuable information on heterogeneities within the network. Two major studies have attempted to gather such egocentric information: the NATSAL studies of sexual contacts in the UK [13–16], and the POLYMOD study of social interactions within 8 European countries [17]. The key to generating a network from such data is to probabilistically assign each alter a set of contacts drawn from the information available from egos; in essence, using the ego data to perform the next step in the snowball sampling algorithm. The simplest way to do this is to generate multiple copies of all the egos and to consider the contacts from each ego to be “half-links”; the half-links within the network can then be connected at random generating a configuration network [18–20]; if more information is available on the status (age, gender, etc.) of the egos and alters then this can also be included and will reduce the set of half-links that can be joined together. However, in the vast majority of modelling studies, the egocentric data have simply been used to construct WAIFW (who-acquires-infection-from-whom) matrices [15, 17, 21] that inform about the relative levels of transmission between different groups (e.g., based on sexual activity or age) but neglect the implicit network properties. This matrix-based approach is often reliable: for STIs it is the extreme heterogeneity in the number of contacts (which are close to being power-law or scale-free distributed; see Section 3.2) that drives the infection dynamics [22] although larger-scale structure does play a role [23]; for social interactions, it is the assortativity between (age-) groups that controls the behaviour, with the number of contacts being distributed as a negative binomial [17]. The POLYMOD matrices have therefore been extensively used in the study of the H1N1 pandemic in 2009, providing important information about the cost-effective vaccination of different age-classes [21, 24].

The general configuration model approach of randomly linking together “half-links” from each ego [18, 19] has been adopted and modified to consider the spread of STIs. In particular, simulations have been used to consider the importance of concurrency in sexual networks [25, 26], where concurrency is defined as being in two active sexual partnerships at the same time. A dynamic sexual network was simulated, with partnerships being broken and reformed such that the network density remained constant over time. The likelihood of two nodes forming a partnership depended on their degree, but this relationship could be tuned to make concurrency more or less common and to make the mixing assortative or disassortative based on the degrees of the two nodes. Transmission of an STI (such as gonorrhoea and chlamydia [25] or HIV [26]) was then simulated upon this dynamic network, showing that increasing concurrency substantially increased the growth rate during the early phase of an epidemic (and, therefore, its size after a given period of time). This greater growth rate was related to the increase in giant component size (see Section 3.1) that was caused by increased concurrency.

A slightly more general approach to the generation of model sexual networks was employed by Ghani et al. [27]. In their network model, individuals had a preferred number of concurrent partners and duration of partnerships, and their level of assortativity was tunable. A gonorrhoea-like infection was simulated on the resulting dynamic network. Regression models were used to consider the association between network structures (either snapshots of the state of the network at the end of simulation or accumulated over the last 90 days of simulation) and prevalence of infection. These simulations showed that increasing levels of concurrent partnerships made invasion of the network more likely and also that the mixing patterns of the most sexually active nodes were most important in determining the final prevalence of infection within the population [27]. The same model was later used to consider the importance of different structural measures and sampling strategies, showing that it was important to endeavour to identify infected individuals with a high number of sexual partners in order to correctly define the high-risk group for interventions [23].

The alternative approach of simulating the behaviour of individuals is obviously highly complex and fraught with a great deal of uncertainty. Despite these problems, three groups have attempted just such an approach: Longini's group at Emory [28–31], Ferguson's group at Imperial [32, 33], and Eubank's group at Los Alamos/Virginia Tech [34, 35]. The models of both Longini and Ferguson are primarily agent-based models, where individuals are assigned a home and work location within which they have frequent infection-relevant contacts together with more random transmission in their local neighbourhood. The Longini models separate the entire population into subunits of 2000 individuals (for the USA) or 13000 individuals (for South-East Asia) who constitute the local population where random transmission can operate; in contrast, the Ferguson models assign each individual a spatial location and random transmission occurs via a spatial kernel. In principle, both of these models could be used to generate an explicit network model of possible contacts. The Eubank model is also agent-based aiming to capture the movements of 1.5 million people in Portland, Oregon, USA; but these movements are then used to define a network based on whether two individuals occur in the same place (there are 180 thousand places represented in the model) at the same time. It is this network that is then used to simulate the spread of infection. While in principle this Eubank model could be used to define a temporally varying and real-valued network (where the strength of connection would be related to the type of mixing in a location and the number of people in the location); in the epidemiological publications [35], the network is considered as a static contact network in which extreme heterogeneity in numbers of contacts is again predicted, and the network has “small world” like properties (see below). A similar approach of generating artificial networks of individuals for stochastic simulations of respiratory disease has been recently applied to influenza at the scale of the United States, and the software made generally available [36]. This software took a more realistic dynamic network approach and incorporated flight data within the United States, but was sufficiently resource-intensive to require specialist computing facilities (a single simulation taking around 192 hours of CPU time). All three models have been used to consider optimal control strategies, determining the best deployment of resources in terms of limiting transmission associated with different routes. The predicted success of various control strategies, therefore, critically depends on the strength of contacts within home, at work, within social groups, and that occuring at random.

Whilst smallpox has been eradicated, concern remains about the possibility of a deliberate release of the disease. The stochastic simulation models of the Longini group have predominantly focused on methods of controlling this infection [28, 31]. Their early work utilised networks of two thousand people with realistic age, household size, and school attendance distributions, with the likelihood of each individual becoming infected being derived from the number and type of contacts with infectious individuals [28]. This paper focused on the use of vaccination to contain a small-scale outbreak of smallpox and concluded that early mass-vaccination of the entire population was more effective than targeted vaccination if there was little or no immunity in the population. Later models [31] combined these subnetworks of two thousand people into a larger network of fifty thousand people (with one hospital), and the adult population were able to contact each other through workplaces and high schools. Here, the focus was on surveillance and containment which were generally concluded to be sufficient to control an outbreak. The epidemiological work of the Eubank group has also focused on a release of smallpox although these simulations showed that encouraging people to stay at home as soon as they began to feel unwell was more important than choice of vaccination protocol [35]; this may in part be attributed to the scale-free structure of the network and hence the superspreading nature of some individuals. 

The Ferguson models have primarily been used to consider the spread and control of pandemic influenza, examining its potential spread from an initial source in South-East Asia [32] and its spread in mainland USA and Great Britain [33]. The models of South-East Asia were primarily based on Thailand, and included demographic information and satellite-based spatial measures of population density. It focused on containment by the targeted use of antiviral drugs and suggested that as long as the reproductive ratio (*R*_0_) of a novel strain was below 1.8, it could be contained by the rapid use of targeted antivirals and social distancing. However, such a strategy could require a stockpile of around 3 million antiviral doses. The models based on the USA and Great Britain, considered a wider range of control measures, including school closures, household prophylaxis using antiviral drugs, and vaccination, and predicted the likely impact of different policies.

### 2.4. Movement Networks

An alternative source of network information comes from the recorded movements of individuals. Such data frequently describe a relatively large network as information on movements is often collected by national or international bodies. The network of movements, therefore, has nodes representing locations (rather than individuals) and edges weighted to capture the number of movements from one location to another—as such the network is rarely symmetric. Four main forms of movement network have played important roles in understanding the spread of infectious diseases: the airline transportation network [37, 38], the movement of individuals to and from work [39, 40], the movement of dollar bills (from which the movement of people can be inferred) [41], and the movement of livestock (especially cattle) [30, 42]. While the structure of these networks has been analysed in some detail, to develop an epidemiological model requires a fundamental assumption about how the epidemic progresses within each locations. All the examples considered in this section make the simplifying assumption that the epidemic dynamics within each location are defined by random (mean-field) interactions, with the network only informing about the flow of individuals or just simply the flow of infection between populations—such a formulation is known as a metapopulation model [43].

Probably the earliest work using detailed movement data to drive simulations comes from the spread of 1918 pandemic influenza in the Canadian Subarctic, based on records kept by the Hudson's Bay Company [44]. A conventional SIR metapopulation model was combined with a network model (the nodes being three fur trading posts in the region: God's Lake, Norway House, and Oxford House), where some individuals remained in their home locations whilst others moved between locations, based on records of arrivals and departures recorded in the post journals. Whilst this model described only a small population, it was able to be parameterised in considerable detail due to the quality of demographic and historical data available and showed that the movement patterns observed interacted with the starting location of a simulated epidemic to change the relative timings of the epidemics in the three communities, but not the overall impact of the disease.

The movement of passenger aircraft as collated by the International Air Transport Association (IATA) provides very useful information about the long-distance movement of individuals and hence how rapidly infection is likely to travel around the globe [37, 45, 46]. Unlike many other network models which are stochastic individual-level simulations, the work of Hufnagel et al. [37] and Colizza et al. [45] was based on stochastic Langevin equations (effectively differential equations with noise included). The early work by Hufnagel et al. [37] focused on the spread of SARS and showed a remarkable degree of similarity between predictions and the global spread of this disease. This work also showed that extreme sensitivity to initial conditions arises from the structure of the network, with outbreaks starting in different locations generating very different spatial distributions of infection. The work of Colizza was more focused towards the spread of H5N1 pandemic influenza arising in South-East Asia and its potential containment using antiviral drugs. However, it was H1N1 influenza from Mexico that initiated the 2009 pandemic, but again, the IATA flight data provided a useful prediction of the early spread [47, 48]. While such global movement networks are obviously highly important in understanding the early spread of pathogens, they unfortunately neglect more localised movements [49] and individual-level transmission networks. However, recent work has aimed to overcome this first issue by including other forms of local movement between populations [40, 50]. This work has again focused on the spread of influenza, mixing long-distance air travel with shorter range commuter movements and with the model predictions by Viboud et al. [40] showing good agreement with the observed patterns of seasonal influenza. An alternative form of movement network has been inferred from the “Where's George” study of the circulation of dollar bills in the USA [38]; this provided far more information about short-range movements, but again did not really inform about the interaction of individuals.

A wide variety (and in practice the vast majority) of movements are not made by aircraft but are regular commuter movements to and from work. The network of such movements has also been studied in some detail for both the UK and USA [39, 40, 51]. The approaches adopted parallel the work done using the network of passenger aircraft, but operate at a much smaller scale, and again, influenza and smallpox have been the considered pathogens. As with the aircraft network certain locations act as major hubs attracting lots of commuters every day; however, unlike the aircraft network, there is the tendency for the network to have a strong daily signature with commuters moving to work during the day but travelling home again in the evening [52]. As such the commuter network can be thought of as heterogeneous, locally clustered, temporal, and with each contact having different strengths (according to the number of commuters making each journey); however, to provide a complete description of population movement, and hence disease transmission requires other causes of movement to be included [51] and requires strong assumptions to be made about individual-level interactions. The key question that can be readily addressed from these commuter-movement models is whether a localised outbreak can be contained within a region or whether it is likely to spread to other nodes on the network [39].

Undoubtedly, one of the largest and most comprehensive data sets of movements between locations comes from the livestock tracing schemes run in Great Britain and being adopted in other European countries. The Cattle Tracing Scheme in particular is spectacularly detailed, containing information of the movements of all cattle between farms in Great Britain; as such, this scheme generates daily networks of contacts between over 30,000 working farms in Great Britain [42, 53–56] (Figure 1(f)). Similar data also exist for the movement of batches of sheep and pigs [57] although here the identity of individual animals making each movement is not recorded. This data source has several key advantages over other movement networks: it is dynamic, in that movements are recorded daily; the movement of livestock is one of the major mechanisms by which many infections are transferred between farms, and the metapopulation assumption that cattle mix homogeneously within a farm is highly plausible. In principle, the information in the Cattle Tracing Scheme can be used to form an even more comprehensive network, treating each cow as a node and creating an edge if two cows occur within the same farm on the same day—this would generate an individual-level network for each day which can then be used to simulate the spread of infection [52].

The early spread of foot and mouth disease (FMD) in 2001 was primarily due to livestock movements, particularly of sheep [58]. Motivated by this epidemic, Kiss et al. [57] conducted short simulated outbreaks of FMD on both the sheep movement network based on 4 weeks' movements starting on 8 September 2004 and simulated synthetic networks with the same degree distribution. Due to the short time-scales considered (the aim being to model spread of FMD before it had been detected), nodes were susceptible, exposed or infected but never recovered, and network connections remained static. Simulated epidemics were smaller on the sheep movement network than the random networks, most likely due to disassortative mixing in the sheep movement network. Similarly, Natale et al. [59] employed a static network simulation of Italian cattle farms. Here, farms were not merely represented as nodes, but a deterministic SI system of ODEs was used to model infection on each node essentially generating a metapopulation model. The only stochastic part of the model was the number of infectious individuals moved between connected farms in each time step. This simulation model highlighted the impact of the centrality of seed nodes (measured in several different ways) upon the subsequent epidemics' course.

The use of static networks to model the very dynamic movement of livestock is questionable. Expanding on earlier work, Green et al. [53] simulated the early spread of FMD through movement of cattle, sheep, and pigs. Here, the livestock network was treated dynamically, with infection only able to propagate along edges on the day when that edge occurred; additional to this network spread, local transmission could also occur. These simulations enabled regional patterns of risk to a new FMD incursion to be assessed, as well as identifying markets as suitable targets for enhanced surveillance. Vernon and Keeling [55] considered the relationship between epidemics predicted from dynamic cattle networks and their static counterparts in more detail. They compared different network representations of cattle movement in the UK in 2004, simulating epidemics across a range of infectivity and infectious period parameters on the different network representations. They concluded that network representations other than the fully dynamic one (where the movement network changes every day) fail to reproduce the dynamics of simulated epidemics on the fully dynamic network.

### 2.5. Contact Tracing Networks

Contact tracing and hence the networks generated by this method can take two distinct forms. The first is when contact-tracing is used to initiate proactive control. This is often the case for STIs, where identified cases are asked about their recent sexual partners, and these individuals are traced and tested; if found to be infected, then contact tracing is repeated for these secondary cases. Such a process is related to the snowball sampling that was discussed earlier, with the notable exception that tracing is only performed from known cases. Similar contact-tracing may operate for the early stages of an airborne epidemic (as was seen for the 2009 H1N1 pandemic), but here, the tracing is not generally iterative as contacts are generally traced and treated so rapidly that they are unlikely to have generated secondary cases. An alternative form of contact-tracing is when a transmission pathway is sought between all identified cases [1, 60, 61]. This form of contact tracing is likely to become of ever-increasing importance in the future when improved molecular techniques and statistical inference allow infection trees to be determined from genetic differences between samples of the infecting pathogen [62].

These forms of network have two main advantages but one major disadvantage. The network is often accompanied by test results for the individuals within the network, as such we not only have information on the contact process but also on the resultant transmission of infection. In addition, when contact tracing is only performed to define an infection tree, there is the added advantage that the infection process itself defines the network of contacts, and hence there is no need for human interpretation of which forms of contact may be relevant. Unfortunately, the reliance on the infection process to drive the tracing means that the network only reflects one realisation of the epidemic process and, therefore, may ignore contacts that are of potential importance and would be needed if the epidemic was to be simulated; therefore, while they can inform about past outbreaks, they have little predictive power.

### 2.6. Surrogate Networks

Obtaining large-scale and reliable information on who contacts whom is obviously very difficult; therefore, there is a temptation to rely on alternative data sets, where network information can be extracted far more easily, and where the data is already collected. As such the movement networks and contact tracing networks discussed above are examples of such surrogate networks although their connection to the physical processes of infection transmission are far more clear. Other examples of networks abound [22, 63–65]; while these are not directly relevant for the spread of infection, they do provide insights into how networks form and grow—structures that are commonly seen in surrogate networks are likely to arise in the types of network associated with disease transmission. One source of network information that would be fantastically rich and also highly informative (if not immediately relevant) is the network of friendships and contacts on social networking sites (such as Facebook); some sites have made data on their social networks available, and these data have been used to examine a range of sociological questions about online interactions [66].

### 2.7. Theoretical Constructs

Given the huge complexity involved in obtaining large-scale and reliable data on real-transmission networks many researchers have instead relied on theoretically constructed networks. These networks are usually highly simplified but aim to capture some of the known (or postulated) features of real-transmission networks—often the simplifications are so extreme that some analytical traction can be gained. Here, we briefly outline some of the commonly used theoretical networks and identify which features they capture; some of the results of how infection spreads on such networks are discussed more fully in Section 4.2.

#### 2.7.1. Configuration Networks

One of the simplest forms of network is to allow each individual to have a set of contacts that it wishes to make (in more formal language each node has a set of half-links), these contacts are then made at random with other individuals based on the number of contacts that they wish to make (half-links are randomly connected) [19]. This obviously creates a network of contacts (Figure 1(c)). The advantage of these configuration networks is that because they are formed from many randomly connected individuals, there are no short loops within the network and a range of theoretical results can be proved ranging from conditions for invasion [18, 67, 68] to descriptions of the temporal dynamics [69]. Unfortunately, the elements that make these networks amenable to theoretical analysis—the lack of assortativity, short loops or clustering—are precisely factors that are thought to be important features of real networks.

An alternative formulation that offers a compromise between tractability and realism occurs when individuals that exist in fully interconnected cliques have randomly assigned links within the entire population [69, 70] (Figure 1(d)). As such, these networks mimic the strong interactions within families and the weaker contacts between them. While such models offer a significant improvement over configuration networks and capture the known importance of the household in transmission, they make no allowance for clustering between households due to spatial proximity. Hierarchical metapopulation models [71] allow for this form of additional structure, where households (or other groupings) are themselves grouped in an ascending hierarchy of clustering.

#### 2.7.2. Lattices and Small Worlds

Both lattice networks and small world networks begin with the same formulation: individuals are regularly spaced on a grid (usually in just one or two dimensions), and each individual is connected to their *k* nearest neighbours—these connections define a lattice. The advantage of such networks is that they retain many elements of the initial spatial arrangement of points, and hence contain both many short loops as well as the property that infection tends to spread locally. There is a clear link between such lattice-based networks and the field of probabilistic cellular automata [72, 73]. The fundamental difficulty with such lattice models is that the presence of short loops and localised spread means that is it difficult (if not impossible) to prove exact results, and hence large-scale multiple simulations are required.

Small world networks improve upon the rigid structure of the lattice by allowing a low number of random contacts across the entire space (Figure 1(e)). Such long range contacts allow infection to spread rapidly though the population and vastly reduce the shortest path length between individuals [74]—this is popularly known as six degrees of separation from the concept that any two individuals on the planet are linked through at most six friends or contacts [75]. Therefore, small world networks offer a step towards reality, capturing the local nature of transmission and the potential for long-range contacts [76, 77]; however, they suffer from neglecting heterogeneity in the number of contacts and the tight clustering of contacts within households or social settings.

#### 2.7.3. Spatial Networks

Spatial networks, as the name suggests, are generated using the spatial location of all individuals in the population, as such lattices and small worlds are a particular form of spatial network. The general methodology initially positions each individual *i* at a specific location ${\underline{x}}_{i}$, usually; these locations are chosen at random, but clustered spatial distributions have also been used [78]. Two individuals (say *i* and *j*) are then probabilistically connected based upon the distance between them; the probability is given by a connection kernel which usually decays with distance such that connections are predominantly localised. These spatial networks (especially when the underlying distribution of points is clustered) have many features that we expect from disease networks although it is unclear if such simple formulations can be truly representative.

#### 2.7.4. Exponential Random Graphs

In recent years, there has been growing interest in exponential random graph models (ERGMs) for social networks, also called the p* class of models. ERGMs were first introduced in the early 1980s by Holland and Leinhardt [79] based on the work of Besag [80]. More recently, Frank and Strauss studied a subset of those that have the simple property that the probability of connection between two nodes is independent of the connection between any other pair of distinct nodes. [81]. This allows the likelihood of any nodes being connected to be calculated conditional on the graph having certain network properties. Techniques such as Markov Chain Monte Carlo can then be used to create a range of plausible networks that agree with a wide variety of information collected on network structures even if the complete network is unknown [82, 83]. Due to their simplicity, ERGMs are widely used by statisticians and social network analysts [84]. Despite significant advances in recent years (e.g., [85]), ERGMs still suffer from problems of degeneracy and computational intractability for large network sizes, which has limited their use in epidemic modelling.

### 2.8. Expected Network Properties

Here, we have shown that a wide variety of network structures have been measured or synthesised to understand the spread of infectious diseases. Clearly, with such a range of networks, no clear consensus can be drawn on the types of underlying network structures that are generally present; in part, this is because different studies have focused on different infectious diseases and different diseases require different transmission routes. However, three factors emerge that are key components of epidemiological networks: heterogeneity in the number of contacts such that some individuals are at a higher risk of both catching and transmitting infection, clustering of contacts such that groups of individuals are often highly interconnected, and some reflection of spatial separation such that contacts usually form locally, but occasional long-range connections do occur. 

Three fundamental problems still exist in the study of networks. Firstly, are there relatively low-dimensional ways of capturing key aspects of a network's structure? What constitutes a key aspect will vary with the problem being studied, but for epidemiological applications, it should be hoped that a universal set of network characteristics may emerge. There is then the task of assessing reasonable and realistic ranges for these key variables based on values computed for known transmission networks—unfortunately very few transmission networks have been recorded in any degree of detail although modern electronic devices may simplify the process in the future. Secondly, there is the related statistical problem of inferring plausible complete networks from the partial information collected by methods such as contact tracing. This is equivalent to seeking an underlying model for the network connections that is consistent with the known partial information, and hence, has strong resonance with the more mechanistically motivated models in Section 2.3. Even when the network is fully realised (and an epidemic observed), there is considerable statistical difficulty in attributing risk to particular contact types. Finally, there are the key questions of predicting the dynamics of infection on any given network—and while for many complex networks, direct simulation is the only approach, for other simplified networks some analytical traction can be achieved, which helps to provide more generic insights into which elements of network structure are most important. These three key areas are discussed below.

## 3. Network Properties

Real networks can exhibit staggering levels of complexity. The challenge faced by researchers is to try and make sense of these structures and reduce the complexity in a meaningful way. In order to make any sense of the complexities present, researchers over several decades have defined a large variety of measurable properties that can be used to characterise certain key aspects [63, 65, 86]. Here, we describe the definitions of the most important characterisations of complex networks (in our view), and outline their impact on disease transmission models.

### 3.1. Components

In general, networks are not necessarily connected; in other words, all parts of the network are not reachable from all others. The component to which a node belongs is that set of nodes that can be reached from it by paths running along edges of the network. A network is said to have a * giant* component if a single component contains the majority of nodes in the network. In directed networks (one in which each edge has an associated direction), a node has both an in-component from which the node can be reached and an out-component that can be reached from that node. A strongly connected component (SCC) is the set of nodes in the network in which each node is reachable from every other node in the component. 

The concept of a giant component is central when considering disease propagation in networks. The extent of the epidemic is necessarily limited to the number of nodes in the component that it begins in, since there are no paths to nodes in other components. In directed networks, in the case of a single initial infected individual, only the out-component of that node is at risk from infection. More generally, the strongly connected component contains those nodes that can be reached from each other. Members of the strongly connected component are most at risk from infection imported at a random node, since a single introduction of infection will be able to reach all nodes in the component.

### 3.2. Degrees, Distributions, and Correlations

**Figure 2:**
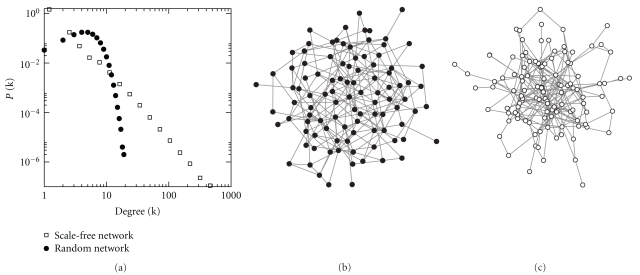
Comparison of random and scale-free networks. (a) Degree distributions for two classes of networks: scale free and random networks. (b) Example random network with 100 nodes and 300 links. All nodes have similar numbers of links. (c) Example scale-free network with 100 nodes and 300 links. Most nodes have few links, with a few nodes having many links.

The *degree* is defined as the *number of neighbours* that a node has and is most often denoted as *k*. In directed graphs, the degree has two components, the number of incoming edges *k* ^in^, (in-degree), and the number of outgoing edges *k* ^out^, (out-degree). The degree distribution is defined as the set of probabilities, *P*(*k*), that a node chosen at random will have degree *k*. Plotting the distribution of degrees of nodes is one of the most basic and important ways of characterising a given network (Figure 2). In addition, useful characterisations are obtained by calculating the moments of the degree distribution. The *n*th moment of *P*(*k*) is defined as

(1) $$\begin{array}{c} \langle k^{n}\rangle =\underset{k}{\sum }k^{n}P\left(k\right), \end{array}$$

with the first moment, 〈*k*〉, being the average degree, the second, 〈*k* ^2^〉 allowing us to calculate the variance 〈*k* ^2^〉−〈*k*〉^2^, and so on. 

The degree distribution is one of the most important ways of characterising a network as it naturally captures the heterogeneity in individuals' potential to become infected as well as cause further infection. Intuitively, the higher the number of edges a node has, the more likely it is to be a neighbour of an already infected node. Also, the more neighbours a node has, the more likely it is to cause a large number of onward cases. Thus, knowing the form of *P*(*k*) is crucial for the understanding of the spread of disease. In random networks of the type studied by Erdös and Rényi, *P*(*k*) follows a binomial distribution, which is effectively Poisson in the case of large networks. Most real social networks have distributions that are significantly different from the random case. 

For the extreme case of *P*(*k*) following an unbounded power law and assuming equal transmission across all edges, Pastor-Satorras and Vespignani [87] showed that the classic result of the epidemic threshold from mean field theory [10] breaks down. In real-transmission networks, the distribution of degree is often heavily skewed, and occasionally follows a power law [22], but is always bounded, leading to the recovery of epidemic threshold, but one which is much lower than expected in evenly mixed populations [88].

The degree distribution provides very useful information on uncorrelated networks such as those produced by configuration models. However, real networks are in general correlated with respect to degree; that is, the probability of finding a node with given degree, *k*, is dependent on the degree of the neighbours of that node, *k*′, which is captured by the conditional probability *P*(*k*′ | *k*). To characterise this behaviour, several measurements have been proposed. The most straightforward, and probably most useful measure, is to consider the average degree of the neighbours of a node

(2) $$\begin{array}{c} k_{nn,i}=\frac{1}{k_{i}}\underset{j\in \text{Nbr}{\text{s}}_{i}}{\sum }k_{j}, \end{array}$$

where the sum of degrees is made over the neighbours (Nbrs) of *i*. One can then calculate the average of *k*_*nn*_ over all nodes with degree *k* which is a direct measure of the conditional probability *P*(*k*′ | *k*), since

(3) $$\begin{array}{c} k_{nn}\left(k\right)=\underset{k^{\prime }}{\sum }k^{\prime }P\left(k^{\prime }\;|\;k\right). \end{array}$$

When *k*_*nn*_(*k*) increases with *k*, the network is said to be assortative on the degree; that is, high-degree nodes have a tendency to link to other high degree nodes, a behaviour often observed in social networks. Other types of networks, such as the internet at router level, show the converse behaviour; that is, nodes of high degree tend to link to nodes with low degree [63, 89].

Characterising degree correlations is important for understanding disease spread. The classic example is the existence of strong correlations in sexual networks which were shown to be a key factor in understanding HIV spread [90]. More recently, mean field solutions of the SIS model on networks have shown that both the speed and extent of an epidemic are dependent on the correlation pattern of the substrate network [91, 92].

### 3.3. Distances

In a network, the *shortest path* between two nodes *i* and *j*, is the path requiring the smallest number of steps to reach *j* from *i*, following edges in the network. There may be (and often there is) more than one shortest path between a pair of nodes. The distance between any pair of nodes *d*_*i*,*j*_ is the minimal number of steps required to reach *j* from *i*, that is, the number of steps in the shortest path. The average distance, 〈*d*〉 is the mean of the distances between all pairs of nodes and measures the typical distance between nodes

(4) $$\begin{array}{c} \langle d\rangle =\frac{1}{N\left(N+1\right)}\underset{i\neq j}{\sum }d_{i,j}, \end{array}$$

where *N* is the number of nodes in the network. The diameter of the network is defined as the maximum shortest path distance between a pair of nodes in the network, max(*d*_*i*,*j*_), which measures the most extreme separation of any two nodes in the network.

Characterising networks in terms of the number of steps needed to reach any node from any other is also important. Real networks frequently display the small-world property; that is, the vast majority of nodes are reachable in a small number of steps. This has clear implications for disease spread and its control. Percolation approaches have shown that the effects of the small-world phenomenon can be profound [93]. If it only takes a short number of steps to reach everyone in the population, diseases are able to spread much more rapidly. 

The notion of shortest distance through a network can be used to quantify how central a given node is in the network. Many measures have been used [94], but the most relevant of these is *betweenness* centrality. Betweenness captures the idea that the more shortest paths pass through a node, the more central it is in the network. So, betweenness is simply defined as the * proportion* of shortest paths that pass through a single node

(5) $$\begin{array}{c} B_{i}=\frac{\#\;\text{shortest}\;\text{paths}\;\text{through}\;i}{N\left(N-1\right)}, \end{array}$$

where *N* is the number of nodes in the network and the denominator quantifies the total number of shortest paths in the network. In terms of disease spread, identifying those nodes with high betweenness will be important. Central nodes are likely to become infected early on in the epidemic, and are also key targets for intervention [3].

### 3.4. Clustering

An important example of an observable property of any network is the *clustering coefficient*, *ϕ*, a measure of the *local density* of a graph. In social network terms, this quantifies the likelihood that the friend of your friend is also your friend. It is defined as the probability that two neighbours of a node will also be neighbours of each other and can be expressed as follows:

(6) $$\begin{array}{c} \phi =\frac{3\times \#\;\text{of}\;\text{triangles}\;\text{in}\;\text{the}\;\text{network}}{\#\;\text{of}\;\text{connected}\;\text{triples}}, \end{array}$$

where a *connected triple* means a single node with edges to a pair of others. *ϕ* measures the fraction of triples that also form part of a triangle. The factor of three accounts for the fact that each triangle is found in three triples and guarantees that 0 ≤ *ϕ* ≤ 1 (and its inclusion depends on the way that triangles in the network are counted).

Locally, the clustering coefficient for each node, *i*, can be defined as the fraction of triangles formed through the immediate neighbours of *i* [74]

(7) $$\begin{array}{c} \phi_{i}=\frac{\#\;\text{triangles}\;\text{centered}\;\text{on}\;i}{\#\;\text{triples}\;\text{centered}\;\text{on}\;i}. \end{array}$$

The clustering property of networks is essential to the understanding of transmission processes. In clustered networks, rapid local depletion of susceptible individuals plays a hugely important role in the dynamics of spread [95, 96]; for a more analytic treatment of this, see Section 4.2 below.

### 3.5. Subgraphs

Degree and clustering characterise some aspects of network structure at an individual level. Considering distances between nodes provides information about the global organisation of the network. Intermediate scales are also present, and characterising these can help in our understanding of network structure and therefore the dynamics of spread. 

At the simplest level, networks can be thought of being comprised of a collection of subgraphs. The simplest subgraph, the *clique*, is defined as a group of more than two nodes where all the nodes are connected to each other by means of edges in both directions. In other words, a clique is a fully connected subgraph, with the smallest example being a triangle. This is a strong definition and one which is only fulfilled in a limited number of cases, most notably households (see Figure 1(d), Section 4.2 and House and Keeling [70]). *n-cliques* relax the above constraint while retaining its basic premise. The shortest path between all the nodes in a clique is one. Allowing this distance to take higher values, one arrives at the definition of * n-cliques*, which are defined as a subgroups of the graph containing more than two nodes where the maximum shortest path distance between any two nodes in the group is *n*. Over the years, many variants of these basic ideas have been formalised in the social network literature and a good summary can be found in Wasserman and Faust [94].

Considering higher order structures can be very informative but is more involved. Milo and coworkers began by looking for specific patterns of connections between nodes in small subgraphs, dubbed *motifs*. Given a connected subgraph of size 3, for example, there are 13 possible motifs. Statistically, some of these appear more often and are found to be overrepresented in certain real networks compared to random networks [97]. Understanding the motif composition of a complex network has been shown to improve the predictive power of deterministic models of transmission when motifs are explicitly modelled (see Section 4.2 and House et al. [98]).

In the above definitions, a subgraph has been defined only in reference to itself. A different approach is to compare the number of internal edges to the number of external edges, arising from the intuitive notion that a *community* will be denser in terms of edges than its surroundings. One such definition, the definition of community in the *strong* sense, is defined as a subgraph in which each node has more edges to other nodes within the subgraph than to any other nodes in the network. Again, this definition is quite restrictive, and in order to relax these constraints, the most commonly used (and most intuitive) definition of communities is groups of nodes that have a high density of edges within them and a lower density of edges between groups. This intuitive definition is behind the most widely used approach for studying community structure in networks. Newman and Girvan formalised this in terms of the * modularity* measure *Q* [99]. Given a particular network which is partitioned into communities, the modularity measure compares the expected number of edges within communities to the actual number of edges within communities.

Although the impact of communities in transmission processes has not been fully explored, a few studies have shown it can have a profound impact on disease dynamics [100, 101]. An alternative measure of how “well-knit” a graph is, named conductance [102], most widely used in the computer science literature has also been found to be important in a range of networks [103].

### 3.6. Higher Dimensional Networks

All of the above definitions have concentrated on networks where the edges remain unchanged over time and all edges have equal weight. Both of these constraints can naturally be relaxed, but generally, this calls for a higher-dimensional characterisation of the edges within the network. It is a matter of common experience that social interactions which can lead to infection do change, with some contacts being repeated regularly, while others are more sporadic. The frequency, intensity, and duration of contacts are all time-varying. How these inherently dynamic networks are represented for the purposes of modelling can have a significant impact on the model outcomes [55, 104]. However, capturing the structure of such dynamic networks in a parsimonious manner remains a substantial challenge. More work has been done on weighted networks, as these are a more straightforward extension of the classical presence-absence networks [105, 106]. In terms of disease spread, the movement networks discussed in Section 2.4 are often considered as weighted [37, 40, 107].

In the sections that follow, we discuss how these network properties can be inferred statistically and the improvements in our understanding of the transmission of infection in networks that have come as a result.

## 4. Model Formulation

### 4.1. Techniques for Simulation

One of the key advantages of the simulation of disease processes on networks is that it enables the study of systems that are too complex for analytical approaches to be tractable. With that in mind, it is worth briefly considering efficient approaches to disease simulation on networks.

There are two main types of simulation model for infectious diseases on networks: discrete-time and continuous-time models; of these, discrete-time simulations are more common, so we discuss them first. In a discrete-time simulation, at every time step, disease may be transmitted along every edge from an infectious node to a susceptible node with a particular probability (which may be the same for all extant edges or may vary according to properties of the two nodes or the edge). Also, nodes may recover (becoming immune, or reverting to being susceptible) during each time-step. Within a time-step, every infection and recovery event is assumed to occur simultaneously. In a dynamic network simulation, the network is typically updated every time step—for example, in a livestock movement network, during time-step *x*, infection could only transmit down edges that occurred during time-step *x*. Clearly, in a directed network, infection may only transmit in the direction of an edge.

Whilst algorithms for discrete-time simulations are not complex, some simple implementation techniques (arising from the observation that most networks of epidemiological interest are sparse) can significantly enhance software performance. In a directed network with *N* nodes, there are *N*(*N* − 1) possible edges; in a sparse network with mean node degree *k*, there are *Nk* ≪ *N*(*N* − 1) edges. Accordingly, rather than representing the network as an *N* by *N* array, where the element in each array is 0 if the edge is absent, nonzero otherwise, it is usually more efficient to maintain a list of the neighbours of each node. Then, if a list of infected nodes is maintained during a simulation run, it is straightforward to consider each susceptible neighbour of an infected node in turn and test if infection is transmitted to that node. Additionally, a fast high-quality pseudorandom number generator such as the Mersenne Twister should be used [108]. The “contagion” software package implements these techniques (amongst others) and is freely available [109]. 

The alternative approach to simulating disease processes on networks is to simulate a series of stochastic Markovian events—the continuous-time approach. Essentially, given the state of the system, it is possible to calculate the probability distributions of when possible subsequent events (i.e., recovery of an infectious node or infection of a susceptible node) will occur. Random draws from these distributions are then made to determine which event occurs next, the state of the system updated, and the process repeated. This approach was pioneered by Gillespie to study the dynamics of chemical reactions [110]; it is, however, computationally intensive, so approximations have been developed. The *τ*-leap method [111], where multiple events are allowed to occur during a time period *τ*, is clearly related to the discrete-time formulation discussed above. However, the ability to allow *τ* to vary during a simulation to account for the processes involved [112] has potential benefits.

The continuous-time approach is clearly in closer agreement with the ideal of standard disease models; however, utilising this method may be computationally prohibitive especially when large networks are involved. Discrete-time models may provide a viable alternative for three main reasons. Firstly, as the time steps involved in the discrete-time model become sufficiently small, we would expect the two models to converge. Secondly, inaccuracies due to the discrete-time formulation are likely to be less substantial in network models compared to random-mixing models, providing two events do not occur in the same neighbourhood during the same time step. Finally, the daily cycle of contacts that regulate most of our lives means that using time steps of less than 24 hours may falsely represent the temporal accuracy that can be attributed to any simulation of the real world.

### 4.2. Analytic Methods

In this section, we use the word “analytic” broadly, to imply models that are directly numerically integrable, without the use of Monte Carlo simulation methods, rather than systems for which all results can be written in terms of fundamental functions, of which there are very few in epidemiology. Analytic approaches to transmission of infection on networks fall into three broad categories. Firstly, there are approaches that calculate exact invasion thresholds and final sizes for special networks. Secondly, there are approaches for calculating exact transient dynamics, including epidemic peak heights and times, but again, these only hold in special networks. Finally, there are approaches based on moment closure that are give approximately correct dynamics for a wide class of networks.

Before considering these approaches on networks, it is worth considering what is meant by nonnetwork mixing and showing explicitly how this can derive the standard transmission terms from familiar differential equation models. Nonnetwork mixing can be taken to have one of two meanings: either that every individual in the population is weakly connected to every other (the mean-field assumption), or that an Erdös-Rényi random graph defines the transmission network, depending on context. To see how this determines the epidemic dynamics, we consider a population of *N* individuals, with a homogeneous independent probability *q* that any pair of individuals is linked on the network, which gives each individual a mean number of edges $\bar{n}=q(N-1)$. We then assume that the transmission rate for infection across an edge is *τ* and that the proportion of the population infectious at a time *t* is *I*(*t*); then, the force of infection experienced by an average susceptible in the population is $\bar{n}\tau I(t)\equiv \beta I(t)$. The quantity *β*, therefore, defines a population-level transmission rate that can be interpreted in one of two ways as *N* → *∞*. In the case where the population is assumed to be fully connected, the limit is that *q* is held at unity, and so *τ* is reduced to as *N* is increased to hold *q*(*N* − 1)*τ* constant. In the case where the population is connected on a random graph, *q* is reduced as *N* is increased to hold $\bar{n}$ constant.

In either case, having defined an appropriate population-level transmission rate, a stochastic susceptible-infectious model of transmission is defined through a Markov chain, in which a population with *X* susceptible individuals and *Y* infectious individuals transitions stochastically to a population with *X* − 1 susceptible individuals and *Y* + 1 infectious individuals at rate *βXY*/(*N* − 1). Then, the exact mean behaviour of such a system in the limit *N* → *∞* then has its transmission behaviour captured by

(8) $$\begin{array}{c} \dot{S}=-\beta S\left(t\right)I\left(t\right), \end{array}$$

where *S*, *I* are the proportion of individuals susceptible and infectious, respectively. The mathematical formalism behind deriving such sets of ordinary differential equations from Markov chains is given by Kurtz [113], and a summary of the application of this methodology to infectious disease modelling is given in Diekmann and Heesterbeek [114]. However, it should be clear that (8) is familiar as the basis of all random-mixing epidemiological models.

In the case of exponentially distributed infectious periods and recovery from infection offering long-lasting immunity, the standard *SIR* equations provide an exact description of the mean behaviour of this system. Nevertheless, the existence of waning immunity, a latent period between an individual becoming infected and being able to transmit infection, and nonexponentially distributed recovery periods are also important for epidemiological applications [10, 42, 115]. These can often be incorporated into analytical approaches through the addition of extra disease compartments, which necessitates extra algebraic and computational effort but typically does not require a fundamental conceptual reevaluation. Sometimes, significant additional complexity does not even modify quantitative epidemiological results—for example, regardless of the rate of waning immunity, length of latent period, or infectious period distribution, if the mean infectious period is *T*, then the basic reproductive ratio is

(9) $$\begin{array}{c} R_{0}=\beta T. \end{array}$$

The estimation of this quantity for complex disease histories, from data likely to be available, is considered by Wallinga and Lipsitch [116]. We, therefore, focus on the transmission process, since this is most affected by network structure, and other elements of biological realism typically act at the individual level. An important caveat to this, however, is when an infected individual's level of transmissibility varies over the course of their infectious period, which sets up correlations between the processes of transmission and recovery that pose a particular challenge for analytic work, especially in structured populations, as noted by for example Ball et al. [117].

#### 4.2.1. Exact Invasion

For nonnetwork mixing, the threshold for invasion is given by the basic reproductive ratio *R*_0_, defined as the expected number of secondary infectious cases created by an average primary infectious case in an otherwise wholly susceptible population. In structured populations, this verbal definition is typically altered to be the secondary cases caused by a typical primary case once the dynamical system has settled into its early asymptotic behaviour. As such, the threshold for invasion is *R*_0_ = 1: for values above this, an infection can grow in the population and the disease can successfully invade; for values below it, each chain of infection is doomed to eventual extinction. Values of *R*_0_ can be measured directly during the course of an epidemic by detailed contact tracing; however, there are considerable statistical issues concerning censoring and data quality.

Provided there are no short closed loops in the network, *R*_0_ can be defined through a next-generation matrix

(10) $$\begin{array}{c} K_{km}=\frac{\left[km\right]\left(m-1\right)}{m\left[m\right]}p, \end{array}$$

where *K*_*km*_ defines the number of cases in individuals with *k* contacts from an individual with *m* contacts during the early stages of the epidemic. Here and elsewhere in this section we use square brackets to represent the numbers of different types on the network; hence, [*m*] is the number of individuals with *m* edges in the network and [*km*] is the number of edges between individuals with *k* and *m* contacts, respectively. In addition, *p* is the probability of infection eventually passing across the edge between a susceptible-infectious pair (for Markovian recovery rate *γ* and transmission rate *τ* this is given by *p* = *τ*/(*τ* + *γ*)). The basic reproductive ratio is given by the dominant eigenvalue of the next-generation matrix

(11) $$\begin{array}{c} R_{0}=||\left(K_{km}\right)||. \end{array}$$

This quantity corresponds to the standard verbal definition of the basic reproductive ratio, and correspondingly the invasion threshold is at *R*_0_ = 1.

Once an appreciable number of short closed loops are present in the network, exact threshold parameters can still sometimes be defined, but these typically depart from the standard verbal definition of *R*_0_. For example, Ball et al. [117] consider a branching process on cliques (households) connected to each other through configuration-model edges—cliques are connected to each other at random (Figure 1(d)). By considering the number of secondary cliques infected by a clique with one initial infected individual, a threshold called *R*_∗_ can be defined. (For the configuration-model of households where each household is of the same size and each individual has the same number of random connections outside the household, the threshold *R*_∗_ is given later as (20); however, the methodology is far more general). The calculation of the invasion threshold for the recently defined triangular configuration model [118, 119] involves calculating both the expected number of secondary infectious individuals and triangles rather than just working at the individual level. Trapman [120] deals with how these sort of results can be related to more general networks through bounding. A general feature of clustered networks for which exact thresholds have been derived so far is that there is a local-global distinction in transmission routes, with a general theory of this given by Ball and Neal [121], where an “overlapping groups” and “great circle” model are also analysed. Nevertheless, care still has to be taken in which threshold parameters are mathematically well behaved and easily calculated (e.g [122]).

#### 4.2.2. Exact Final Size

The most sophisticated and general way to obtain exact results for the expected final size of a major outbreak on a network is called the *susceptibility set* argument and the most general version is currently given by Ball et al. [117]. We give an example of these kind of arguments from Diekmann et al. [123], who consider the simpler case of a network in which each individual has *n* contacts. Where there is a probability *p* of infection passing across a given network link (so for transmission and recovery at rates *τ* and *γ*, resp., *p* = *τ*/(*τ* + *γ*)), the probability that an individual avoids infection is given by

(12) $$\begin{array}{c} S_{\infty }={\left(1-p+\tilde{S}p\right)}^{n},\\ \tilde{S}={\left(1-p+\tilde{S}p\right)}^{n-1}. \end{array}$$

Here, a two-step process is needed because in an unclustered, regular graph two generations of infection are needed to stabilise the network correlations and so the auxiliary variable $\tilde{S}$ must also be solved for. Once this and *S*_*∞*_ are known, the expected attack rate is *R*_*∞*_ = 1 − *S*_*∞*_.

#### 4.2.3. Approximate Final Size

The main way to calculate approximate final sizes is given by percolation-based methods. These were reviewed by Bansal et al. [124] and also in [125]. Suppose that we remove a fraction *φ* of links from the network and can derive an expression for the fraction of nodes remaining in the giant component of the network, *f*(*φ*). Then,

(13) $$\begin{array}{c} R_{\infty }\approx f\left(1-p\right), \end{array}$$

and an invasion threshold is given by the value of *p* for which this final size becomes nonzero in the “thermodynamic limit” of very large network size. This approach is not exact for clustered graphs, but for unclustered graphs exact results like (12) are reproduced.

#### 4.2.4. Exact Dynamics

Some of the earliest work on infectious diseases involved the exact solution of master equations (where the probability of the population being in each possible configuration is calculated) on small, fully connected graphs as summarised in Bailey [126]. The rate at which the complexity of the system of master equations grows means that these equations quickly become too complex to integrate for the most general network. The presence of symmetries in the network, however, does mean that automorphism-driven lumping is one way to manipulate the master equations (whilst preserving the full stochastic information about the system) for solution [127]. At present, this technique has only been applied to relatively simple networks; however, there are no other highly general methods of deriving exact lower-dimensional systems of equations from the master equations.

Nevertheless, other specific routes do exist that allow exact systems of equations of lower dimensionality to be derived for special networks. For static networks constructed using the configuration model (where individuals have heterogeneous degree but connections are made at random such that the presence of short loops can be ignored in a large network, see Figure 1(c)), an exact system of equations for *SIR* dynamics in the limit of large network size was provided by Ball and Neal [69]. This construction involves attributing to each node an “effective degree”, which starts the epidemic at its actual degree, and measures connections still available as routes of infection and is, therefore, reduced by transmission and recovery. Using notation consistent with elsewhere in this paper (and ignoring the global infection terms that were included by Ball and coworkers) this yields the relatively parsimonious set of equations

(14) $$\begin{array}{cc} {\dot{S}}_{k} & =-\rho \left(\left(\tau +\gamma \right)kS_{k}-\gamma \left(k+1\right)S_{k+1}\right),\\ {\dot{I}}_{k} & =\tau \left(\left(k+1\right)I_{k}-kI_{k}\right)-\gamma I_{k}\\ & \;+\rho \left(\left(k+1\right)\left(\tau \left(S_{k+1}+I_{k+1}\right)+\gamma I_{k+1}\right)-k\left(\tau +\gamma \right)I_{k}\right),\\ \rho & :=\frac{\sum_{k}^{}kI_{k}}{\sum_{l}^{}l\left(S_{l}+I_{l}\right)}. \end{array}$$

Here, *S*_*k*_, *I*_*k*_ are the proportion of effective degree *k* susceptible and infectious individuals, respectively. Hence, for a configuration-network where the maximum degree is *K*, we require just 2*K* equations to retrieve the exact dynamics.

While *R*_0_ can be derived using expressions like (11), calculation of the asymptotic early growth rate *r* requires systems of ODEs like (14). If we assume that transmission and recovery are Markovian processes with rates *τ* and *γ*, respectively, two measures of early behaviour are

(15) $$\begin{array}{c} R_{0}^{\text{CM}}=\frac{\langle n\left(n-1\right)\rangle }{\langle n\rangle }\frac{\tau }{\tau +\gamma },\\ r^{\text{CM}}=\frac{\langle n\left(n-2\right)\rangle }{\langle n\rangle }\tau -\gamma , \end{array}$$

where 〈·〉 informs about the average over the degree distribution. These quantities tell us that the susceptibility to invasion of a network increases with both the mean and the variance of the degree distribution. This closely echos the results for risk-structured models [10] but with an extra term of −1 due to the network, representing the fact that the route through which an individual acquired infection is closed off for future transmission events.

For more structured networks with a local-global distinction, there are two limits in which exact dynamics can also be derived. If the network is composed of *m* communities of size *n*_1_,…, *n*_*m*_, with the between-community (global) mixing determined by a Poisson process with rate ${\overset{̅}{n}}_{G}$ and the within-community (local) mixing determined by a Poisson process with rate ${\overset{̅}{n}}_{L}$, then in the limit as the communities become large, *n*_*i*_ → *∞*, the epidemic dynamics on the system are

(16) $$\begin{array}{c} {\dot{S}}_{a}=-S_{a}\left(\beta_{L}I_{a}+\alpha \overset{}{\underset{b\neq a}{\sum }}I_{b}\right),\\ {\dot{I}}_{a}=S_{a}\left(\beta_{L}I_{a}+\alpha \overset{}{\underset{b\neq a}{\sum }}I_{b}\right)-\gamma I_{a}. \end{array}$$

where *S*_*a*_ and *I*_*a*_ are the proportion of individuals susceptible and infectious in community *a*, and

(17) $$\begin{array}{c} \alpha =\frac{{\bar{n}}_{G}\tau }{\left(m-1\right)},\;\;\beta_{L}={\bar{n}}_{L}\tau . \end{array}$$

Hence, we have a classic metapopulation model [43], defined in terms of Poisson local and global connections and large local community sizes.

In the limit where ${\bar{n}}_{L}\to (n-1)$ and *m* → *∞*—such that there are infinitely many communities of equal size and each community forms a fully interconnected clique— “self-consistent” equations such as in Ghoshal et al. [128] and House and Keeling [70] are exact. These equations evolve the proportion of cliques with *x* susceptibles and *y* infecteds, *P*_*x*,*y*_, as well as the proportion of infecteds in the population, *I*, as follows:

(18) $$\begin{array}{cc} I & =\frac{1}{n}\underset{x,y}{\sum }yP_{x,y},\\ {\dot{P}}_{x,y} & =\gamma \left(-yP_{x,y}+\left(y+1\right)P_{x,y+1}\right)\\ & \;+\tau \left(-xyP_{x,y}+\left(x+1\right)\left(y-1\right)P_{x+1,y-1}\right)\\ & \;+\beta_{G}I\left(-xP_{x,y}+\left(x+1\right)P_{x+1,y-1}\right), \end{array}$$

where $\beta_{G}={\bar{n}}_{G}\tau$. 

Both of these two local-global models, the metapopulation model (16) and the small cliques model (18), are reasonably numerically tractable for modern computational resources, provided the relevant finite number (*m* or *n*, resp.) is not too large. The basic reproduction number for the first system is clearly

(19) $$\begin{array}{c} R_{0}=\frac{1}{\gamma }\left(\beta_{L}+\left(m-1\right)\alpha \right), \end{array}$$

while for the second, household model, invasion is determined by

(20) $$\begin{array}{c} R_{*}={\bar{n}}_{G}\frac{\tau }{\tau +\gamma }Z_{\infty }^{n}\left(\tau ,\gamma \right), \end{array}$$

where *Z*_*∞*_^*n*^(*τ*, *γ*) is the expected final size of an epidemic in a household of size *n* with one initial infected. Of course, the within- and between-community mixing for real networks is likely to be much more complex than may be captured by a Poisson process, but these two extremes can provide useful insights. These models show that network structure of the form of communities reduces the potential for an infectious disease to spread, and hence, greater transmission rates are required for the disease to exceed the invasion threshold.

#### 4.2.5. Approximate Dynamics

While all the exact results above are an important guide to intuition, they only hold for very specialised networks. A large class of models exists that form a bridge between “mean-field” models and simulation by using spatial or network moment closure equations. These are highly versatile models. In general, invasion thresholds and final sizes can be calculated rigorously, but exact calculation of transient dynamics is only possible for very special networks. If one wants to calculate transient effects in general network models—most importantly, peak heights and times—then moment closure is really the only versatile way of calculating desired quantities without relying on full numerical simulation.

It is also worth noting that there are many results derived through these “approximate” approaches that are the same as exact results or are numerically indistinguishable from exact results and simulation. We give some examples below and also note that the dynamical PGF approach [129] is numerically indistinguishable from the exact model (14) above for certain parameter values [130]. What is currently lacking is a rigorous mathematical proof of exactness for ODE models other than those outlined in Section 4.2.4 above. While for many practical purposes the absence of such a proof will not matter, we preserve here the conceptual distinction between results that are provably exact, and those that are numerically exact in all cases tested so far.

The idea of moment closure is to start with an exact but unclosed set of equations for the time evolution of different units of structure. Here, we show how these can be derived by considering the rates of change of both types of individual and types of connected pair. Such pairwise moment closure model are a natural extension to the standard (random-mixing) models, given that infection is passed between pairs of infected individuals

(21) $$\begin{array}{cc} \left[\dot{S_{\kappa }}\right] & =-\tau \left[S_{\kappa }⟵I\right],\\ \left[\dot{I_{\kappa }}\right] & =\tau \left[S_{\kappa }⟵I\right]-\gamma \left[I_{\kappa }\right],\\ \left[\dot{S_{\kappa }S_{\lambda }}\right] & =-\tau \left[S_{\kappa }S_{\lambda }⟵I\right]+\left[S_{\lambda }S_{\kappa }⟵I\right],\\ \left[\dot{S_{\kappa }I_{\lambda }}\right] & =\tau \left(\left[S_{\kappa }S_{\lambda }⟵I\right]-\left[I\to S_{\kappa }I_{\lambda }\right]-\left[S_{\kappa }⟵I_{\lambda }\right]\right)-\gamma \left[S_{\kappa }I_{\lambda }\right],\\ \left[\dot{I_{\kappa }I_{\lambda }}\right] & =\tau \left(\left[I\to S_{\kappa }I_{\lambda }\right]+\left[I\to S_{\lambda }I_{\kappa }\right]+\left[S_{\kappa }⟵I_{\lambda }\right]+\left[S_{\lambda }⟵I_{\kappa }\right]\right)\\ & \;-2\gamma \left[I_{\kappa }I_{\lambda }\right],\\ \left[\dot{S_{\kappa }R_{\lambda }}\right] & =-\tau \left[I\to S_{\kappa }R_{\lambda }\right]+\gamma \left[S_{\kappa }I_{\lambda }\right],\\ \left[\dot{I_{\kappa }R_{\lambda }}\right] & =\tau \left[I\to S_{\kappa }R_{\lambda }\right]+\gamma \left(\left[I_{\kappa }I_{\lambda }\right]-\left[I_{\kappa }R_{\lambda }\right]\right). \end{array}$$

Here, we use square brackets to represent the prevalence of different species within the network. We also use some nonstandard notation to present several diverse approaches in a unified framework: generalised indices *κ*, *λ* represent any property of a node (such as its degree), while arrows represent the direction of infection (and so for a directed network, the necessity that an edge in the appropriate direction be present), see Table 1.

Clearly, the system (21) is not closed as it relies on the number of connected triples, and so some form of approximate closure must be introduced to relate the triples to pairs and nodes, which will depend on underlying properties of the network. Most commonly, these closure assumptions deal with heterogeneity in node degree, assortativity, and clustering at the level of triangles. Examples include Keeling [95] and Eames and Keeling [96], where the generalised variables *κ*, *λ* above stand for node degrees (*k*, *l*), the triple closure is symmetric with respect to the direction of infection, and the network is assumed to be static and nondirected. A general way to write the closure assumption is

(22) $$\begin{array}{cc} \left[A_{k}B_{l}C_{m}\right] & \approx \frac{\left(l-1\right)}{l}(\left(1-\phi \right)\frac{\left[A_{k}B_{l}\right]\left[B_{l}C_{m}\right]}{\left[B_{l}\right]}\\ & \;\;\;\;\;+\phi \frac{\bar{n}N}{km}\frac{\left[A_{k}B_{l}\right]\left[B_{l}C_{m}\right]\left[C_{m}A_{k}\right]}{\left[A_{k}\right]\left[B_{l}\right]\left[C_{m}\right]}). \end{array}$$

where $\bar{n}\equiv \langle n\rangle$ is again the average degree distribution and *ϕ* measures the ratio of triangles to triples as a means of capturing clustering within the network (see Section 3.4). The typical way to analyse the closed system is direct numerical integration; however, some analytic traction can be gained. One example is the use of a linearising Ansatz to derive the early asymptotic behaviour of the dynamical system. Interestingly, when this is done for *ϕ* = 0 (such that there are no triangular loops in the network) as in Eames and Keeling [96], the result for the early asymptotic growth rate agrees with the exact result of (10). In [95], the differential equations for an *n*-regular graph were also manipulated to give an expression for final size that agreed with the exact result  (12)

Equation (22), however, is not the only possible network moment closure regime: Boots and Sasaki [131] and Bauch [132] considered regimes in which closure depended on the disease state (i.e., triples composed of different arrangements of susceptibles and infecteds close differently) to deal with spatial lattice-based systems and early disease invasion respectively. For example Boots and Sasaki [131] use a closure where

(23) $$\begin{array}{cc} \left[SOO\right] & \approx \varepsilon \frac{\bar{n}-1}{\bar{n}}\frac{\left[SO\right]\left[OO\right]}{\left[O\right]},\\ \left[IOO\right] & \approx \varepsilon \frac{\bar{n}-1}{\bar{n}}\frac{\left[IO\right]\left[OO\right]}{\left[O\right]},\\ \left[SOS\right] & \approx \left[OS\right]\frac{\bar{n}-1}{\bar{n}}\left(1-\varepsilon \frac{\left[OO\right]}{\left[O\right]}-\frac{\left[IO\right]}{\left[O\right]}\right),\\ \left[ABC\right] & \approx \frac{\bar{n}-1}{\bar{n}}\frac{\left[AB\right]\left[BC\right]}{\left[B\right]}\;\text{for}\;\text{all}\;\text{other}\;\text{triples}, \end{array}$$

where *O* represents empty sites within the network that are not currently occupied by individuals, and the parameter *ε* = 0.8093 accounts for the clustering within lattice-based networks. House and Keeling [133] considered a model of infection transmission and contact tracing on a network, where the closure scheme for [*ABC*] triples was asymmetric in *A* and *C*—this allowed the natural conservation of quantities in a highly clustered system.

The work on dynamical PGF models [129] can be seen as an elegant simplification of this pairwise approach that is valid for SIR-type infection dynamics on configuration model networks. The equations can be reformulated as

(24) $$\begin{array}{c} S=g\left(\theta \right),\\ \dot{I}=\tau p_{I}\theta g^{\prime }\left(\theta \right)-\gamma I,\\ {\dot{p}}_{I}=\tau p_{S}p_{I}\theta \frac{g^{\prime \prime }\left(\theta \right)}{g^{\prime }\left(\theta \right)}-\tau p_{I}\left(1-p_{I}\right)-\gamma p_{I},\\ {\dot{p}}_{S}=\tau p_{S}p_{I}\left(1-\theta \frac{g^{\prime \prime }\left(\theta \right)}{g^{\prime }\left(\theta \right)}\right),\\ \dot{\theta }=-\tau p_{I}\theta , \end{array}$$

where *g* is the probability generating function for the degree distribution, *p*_*S*_ and *p*_*I*_ correspond to the number of contacts of a susceptible that are susceptible or infected, respectively, and *θ* is defined as probability that a link randomly selected from the entire network has not been associated with the transmission of infection. Here, the closure assumption is implicit in the definition of *S*; that is, an individual only remains susceptible if all of its links have not seen the transmission of infection, and that the probability is independent for each link, which is comparable to the assumptions underlying the formulation by Ball and Neal [69], equation (14). The precise link between this PGF formulation and the pairwise approach is discussed more fully in House and Keeling [134].

There are many other extensions of this general methodology that are possible. Writing ODEs for the time evolution of triples and closing at a higher order allows the consideration of the epidemiological consequences of varying motif structure [98]. Sharkey et al. [135] considered closure at triple level on directed networks, which involved a more sophisticated treatment of third-order clustering due to the larger repertoire of three-motifs in directed (as compared to undirected) networks. It is also possible to combine stochastic and network moment closure [136]. Time-varying, dynamical networks, particularly applied to sexually transmitted infections where partnerships vary over the course of an epidemic, were considered using approximate ODE-based models by Eames and Keeling [137] and Volz and Meyers [138]. Sharkey [139] considered models appropriate for local networks with large shortest path lengths, where the generic indices *μ*, *λ* in (21) stand for node numbers *i*, *j* rather than node degrees *k*, *l*.

Another approach is to approximate the transmission dynamics in the standard (mean-field) differential equations models. Essentially, this is a form of moment closure at the level of pairs rather than triples. For example, in Roy and Pascual [140] the transmission rate takes the polynomial form

(25) $$\begin{array}{c} \text{Transmission}\;\text{rate}\;\text{to}\;S_{k}\;\text{from}\;I_{l}\propto kl{\left(S_{k}\right)}^{p}{\left(I_{l}\right)}^{q}, \end{array}$$

where the exponents, *p* and *q*, are typically fitted to simulated data but are thought to capture the spatial arrangement of susceptible and infected nodes. Also, Kiss et al. [141] suggest

(26) $$\begin{array}{c} \text{Transmission}\;\text{rate}\;\text{to}\;S_{k}\;\text{from}\;I_{l}\propto k\left(l-1\right)\left(S_{k}\right)\left(I_{l}\right), \end{array}$$

as a way of accounting for each infected “losing” an edge to its infectious parent.

Finally, a very recent work [142] presents a dynamical system to capture epidemic dynamics on triangular configuration model networks; the relationship between this and other ODE approaches is likely to be an active topic for future work.

This diversity of approaches leads to some important points about methods based on moment closure. These methods are extremely general and can be applied to consider almost any aspect of network structure or disease natural history; they can be applied to populations not currently amenable to direct simulation due to their size, and they do not require a complete description of the network to run—only certain statistical properties. However, there are currently no general methods for the proposal of appropriate closure regimes nor any derivation of the limits on dynamical biases introduced by closure. Therefore, closure methods sit somewhere in between exact results for highly specialised kinds of network and stochastic simulation, where intuitive understanding and general analysis are more difficult.

### 4.3. Comparison of Analytic Models with Simulation

In the papers that introduced them, the differential-equation-based approximate dynamical systems above were compared to stochastic simulations on appropriate networks. Two recent papers making a comparison of different dynamical systems with simulation are Bansal et al. [124] and Lindquist et al. [130]. There are, however, several issues with attempts to compare deterministic models with simulation and also with each other.

Firstly, it is necessary to define what is meant by agreement between a smooth, deterministic epidemic curve and the rough trajectories produced by simulation. Limiting results about the exactness of different ODE models assume that both the number of individuals infectious and the network size are large, and so the early behaviour of simulations, when there are few infectious individuals, is often dominated by stochastic effects. There are different ways to address this issue, but even after this has been done, there are two sources of deviation of simulations from their deterministic limit. The first of these is the number of simulations realised. If there is a summary statistic such as the mean number of infectious individuals over time, then the confidence interval in such a statistic can be made arbitrarily small by running additional simulations, but agreement between the deterministic limit and a given realisation may still be poor. The second source of deviation is the network size. By increasing the number of nodes, the prediction interval within which the infection curve will fall can be made arbitrarily small; however, the computational resources needed to simulate extremely large networks can quickly become overwhelming.

More generally, each approximate model is designed with a different application in mind. Models that perform well in one context will often perform poorly in another, and this means that “performance” of a given model in terms of agreement with simulation will primarily be determined by the discrete network system on which simulations are performed.

**Figure 3:**
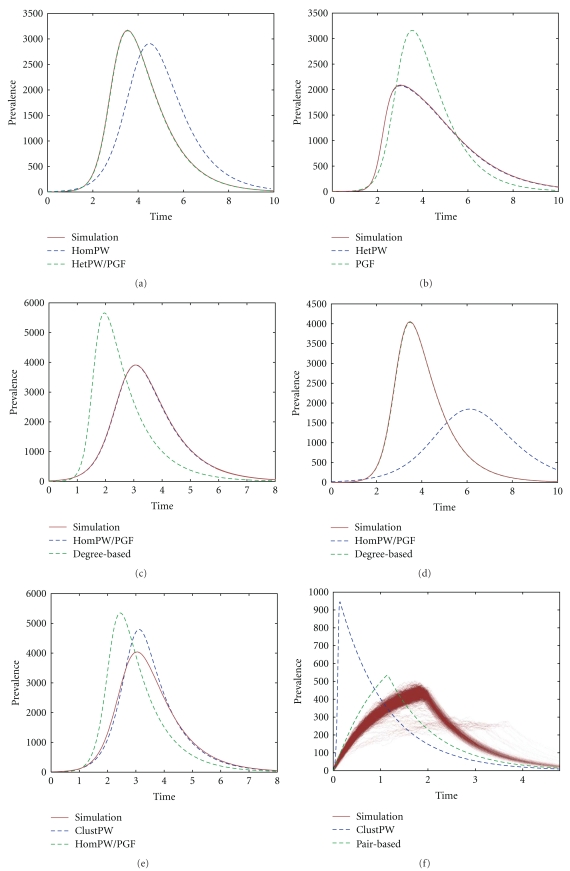
Comparison of simulation and deterministic models for six networks. (a) Two-group configuration model network. (b) Two-group assortative network. (c) Static regular network. (d) Dynamical regular network. (e) Regular clustered network. (f) One-dimensional lattice.

The above considerations motivate the example comparisons with simulation that we show in Figure 3. This collection of plots is intended to show a variety of different example networks, and the dynamical systems intended to capture their behaviour.

In Figures 3(a)–3(e), continuous-time simulations have their temporal origin shifted so that they agree on the time at which a cumulative incidence of 200 is reached, and then confidence intervals in the mean prevalence of infection are achieved through bootstrapping. The 95% confidence interval is shown as a red shaded region (although typically, this is sufficiently narrow it resembles a line). Six different deterministic models are compared to simulations: HomPW is the pairwise model of Keeling [95] with zero clustering, HetPW is the heterogeneous pairwise model of Eames and Keeling [96], ClustPW is the improved clustered pairwise closure of House and Keeling [133], PGF is the model of Volz [129], Pair-based is the model of Sharkey [139], integrated using the supplementary code from Sharkey [143], and Degree-based is the model of Pastor-Satorras and Vespignani [87].

Figure 3(a) shows a heterogeneous network composed of two risk groups, constructed according to the configuration model [18]. In this case, models that incorporate heterogeneity like HetPW and PGF (which are numerically indistinguishable in this case and several others) are in very close agreement with simulation, while just taking the average degree as in HomPW is a poor choice. In Figure 3(b), assortativity is added to the two group model following the approach of Newman [89], and HetPW outperforms PGF. Figures 3(c) and 3(d) show regular graphs with four links per node, but while Figure 3(c) is static in Figure 3(d) the rate of making and breaking links is much faster than the epidemic process. Models like HomPW and PGF are therefore better for the former and degree-based models are better for the latter—in reality the ratio of the rate of network change to the rate of transmision may not be either large or small and so a more sophisticated method may be best [137, 138]. Figure 3(e) shows a graph with four links per node where clustering has been introduced by the rewiring method of Bansal et al. [144] sometimes called the “big V” [133]. In this case, ClustPW performs better than HomPW and PGF, but clearly there is significant inaccuracy around the region of peak prevalence and so this model captures qualitatively the effects of clustering without appearing to be exact for this precise network. Finally, Figure 3(f) considers the case of a one-dimensional next-nearest-neighbour lattice (so there are four links per node). This introduces long path lengths between nodes in addition to clustering, meaning that the system does not converge onto a period of asymptotic early growth and so realisations are shown as a density plot rather than a confidence interval. ClustPW accounts for clustering but not long path lengths and so is in poor agreement with simulation while the pair-based curve captures the qualitative behaviour of an epidemic on this lattice whilst being quantitatively a reasonable approximation.

## 5. Inference on Networks

In order to be predictive, epidemic models rely on valid values for parameters governing outbreak dynamics, conditional on the population structure. However, obtaining these parameters is complicated by the fact that even when knowing the underlying contact network structure, infection events are censored—it is only when disease is detected either from symptoms or laboratory tests that a case becomes apparent. In attempting to surmount this difficulty, parameter estimates are often obtained by making strong assumptions as to the infectious period or through ad hoc methods with unknown certainty. Measuring the uncertainty in such estimates is as important as obtaining the estimates themselves in providing an honest risk prediction. Given these difficulties, inference for epidemic processes has perhaps received little attention in comparison to its simulation counterpart. 

The presence of contact network data for populations provides a unique opportunity to estimate the importance of various modes of disease transmission from disease incidence or contact tracing data. For example, given knowledge of the rate of contact between two individuals, it is possible to infer the probability that a contact results in an infection. If data on mere connectivity (i.e., a 1 if the individuals are connected and 0 otherwise) is available, then it is still possible to infer a rate of infection between connected individuals. Thus, the detail of the inference is determined to a large extent by the available detail in the network data [145].

### 5.1. Availability of Data

Epidemic models are defined in terms of times of transitions between infection states, for example a progression from susceptible, to infected, to removed (i.e., recovered with lifelong immunity or dead) in the so-called “SIR” model. Statistical inference requires firstly that observations of the disease process are made: at the very least, this comprises the times of case detections, remembering that infection times are always censored (you only ever know you have a cold a few days after you caught it). In addition, covariate data on the individuals provides structure to the population and begins to enable the statistician to make statements about the importance of individuals' relationships to one another in terms of disease transmission. Therefore, any covariate data, however slight, effectively implies a network structure upon which disease transmission can be superimposed. 

As long as populations are relatively small (e.g., populations of farms in livestock disease analysis), it is common for models to operate at the individual level, providing detailed information on case detection times and perhaps even information on epidemiologically significant historical contact events [146–148]. In other populations, however, such detailed data may not be available due to practical and ethical reasons. Instead, data is supplied on an aggregated spatial and/or temporal basis. For the purposes of inference, therefore, this can be regarded as a household model, with areas constituting households. 

In a heterogeneous population, the behaviour of an epidemic within any particular locality is governed by the relationship between infected and susceptible individuals. For inference in the early stages of an epidemic, it is important to quantify the amount of uncertainty in the underlying contact networks as the early growth of the epidemic is known to be subexponential due to the depletion of the local susceptible population. This contrasts markedly to the exponential growth observed in a large homogeneously mixing population [10]. When the network is known and details of individual infections are available, contact tracing data may be used to infer the network; this data could also be used for inference on the epidemic parameters [116, 149]. Conversely, if the network is completely unknown, it would be useful if estimation of both the epidemic parameters and parameters specifying the structure of the network was possible. This is a difficult problem because the observed epidemic contains very limited information about the underlying network, as demonstrated by Britton and O'Neill [150]. However, with appropriate assumptions, some results can be obtained; the limited amount of existing work in this area is described in Section 5.3.1 below although clearly the problem is worthy of further study.

#### 5.1.1. Inference on Homogeneous Models

For homogeneous models the basic reproduction number, or *R*_0_, has several equivalent definitions and can be defined in terms of the transmission rate *β* and removal rate *γ*. For nonhomogeneous models, the definitions are not equivalent; see for example [122]. 

Although inference for *β* and *γ* is difficult for real applications (see below), it turns out that making inference on *R*_0_ (as a function of *β* and *γ*) is rather more straightforward. Heffernan et al. [151] summarise various methods for estimating *R*_0_ from epidemiological data based on endemic equilibrium, average age at infection, epidemic final size, and intrinsic growth rate [114, 152, 153]. However, these methods all rely on observing a complete epidemic, and hence for real-time analysis during an epidemic, we must make strong assumptions concerning the number of currently undetected infections. An example of inference for *R*_0_ based upon complete epidemic data is provided by Stegeman et al. [154], where data from the 2003 outbreak of High Pathogenicity Avian Influenza H7N7 is fitted to a chain-binomial model using a generalised linear model. 

 Obviously, complete or near-complete epidemic data is rare and hence it is desirable to perform inference based upon partial observation. This is particularly relevant for real time estimation of *R*_0_. For example, Cauchemez et al. [155] attempt to estimate *R*_0_ in real-time by constructing a discrete-time statistical model that imputes the number of secondary cases generated by each primary case. This is based on the method of Wallinga and Teunis [156] who formulate a likelihood function for inferring who infected whom from dates of symptom onset

(27) $$\begin{array}{c} L\left(i\;\text{infected}\;j\right)=\frac{w\left(t_{j}-t_{i}\right)}{\sum_{j\neq k}^{}w\left(t_{j}-t_{k}\right)}, \end{array}$$

where *w*(·) is the probability density function for the *generation interval t*_*j*_ − *t*_*i*_, that is, the time between infector *i*'s infection time and infectee *j*'s infection time. Of course, infection times are never observed in practice so symptom onset times are used as a proxy, with the assumption that the distribution of infection time to symptom onset time is the same for every individual. Bayesian methods are used to infer “late-onset” cases from known “early-onset” cases, but large uncertainty of course remains when inferring the reproductive ratio close to the current time as there exists large uncertainty about the number of cases detected in the near future. Additionally, a model for *w*(·) must be chosen (see, [157]). 

 The tradeoff in the simplicity of estimating *R*_0_ in these ways, however, is that although a population wide *R*_0_ gives a measure of whether an epidemic is under control on a wide-scale, it give no indication as to regional-level, or even individual-level, risk. Moreover, the two examples quoted above do not even attempt to include population heterogeneity into their models though the requirement for its inclusion is difficult to ascertain in the absence of model diagnostics results. It is postulated, therefore, that a simple measure of *R*_0_, although simple to obtain, is not sufficient in order to make tactical control-policy decisions. In these situations, knowledge of both the transmission rate and removal rate are required.

### 5.2. Inference on Household Models

Inference for households models is well developed in comparison to inference for other “network” models. In essence, this is for three main reasons: firstly, it is a reasonable initial approximation to assume that infection either occurs within the home or from a random source in the population. Secondly, entire households can be serologically sampled following an epidemic, such that the distribution of cases in households of given sizes can be ascertained. Finally, it is often a reasonable approximation that following introduction of infection into the household, the within-household epidemic will go extinct before any further introductions—which dramatically simplifies the mathematics.

The first methods proposed for such inference are maximum likelihood procedures based upon chain-binomial models, such as the Reed-Frost model, or the stochastic formulation of the Kermack-McKendrick model considered by Bartlett [158]. These early methods are summarised by Bailey [126]. They, and the significant majority of methods proposed for household inference to date, use final-size data which can be readily obtained from household serology results. A simplifying assumption to facilitate inference in most methods is that the epidemics within the various households evolve independently (e.g., see the martingale method of Becker [159], which requires the duration of a latent period to be substantial for practical implementation).

Additionally, fixed probabilities *p*_*C*_ and *p*_*H*_, corresponding to a susceptible individual escaping community-acquired infection during the epidemic and escaping infection when exposed to a single infected household member, respectively, were initially assumed [160]. Two important, realistic extensions to this framework are to incorporate different levels of risk factors for individuals [161] and to introduce dependence of *p*_*H*_ on an infectious period [162]. The latter inclusion was enabled by appealing to results of Ball et al. [163]. These types of methods are largely based upon the ability to generate closed form formulae for the final size distribution of the models. 

The ability to relax assumptions further has been predominately due to use of Markov chain Monte Carlo (MCMC) methods as first considered by O'Neill et al. [162] for household models following earlier studies of Gibson and Renshaw [164] and O'Neill and Roberts [165] who focused on single, large outbreaks. This methodology has been used to in combination with simulation and data augmentation approaches to tailor inference methods for specific data sets of interest; for example, Neal and Roberts [166] consider a model with a spatial component of distance between households and data containing details of dates of symptoms and appearance of rash and has also resulted in a growing number of novel methods for inference, for example Clancy and O'Neill [167] consider a rejection sampling procedure and Cauchemez et al. [168] introduce a constrained simulation approach. Even greater realism can be captured within household models by considering the different compositions of households and, therefore, the weighted nature of contacts within households. For example, Cauchemez et al. [169] considered household data from the Epigrippe study of influenza in France 1999-2000 and showed that children play a key role in the transmission of influenza and the risk of bringing infection into the household.

Whilst new developments are appearing at an increasing rate, the significant majority of methods are based upon final size data and are developed for SIR disease models, perhaps due in part to the simplification of arguments for deriving final size distributions. One key, but still unanswered question from these analyses of household epidemics is how the transmission rate between any two individuals in the household scales with the total number of individuals in the household (compare Longini and Koopman [160] and Cauchemez et al. [169]). Intuition would suggest that in larger households the mixing between any two individuals is decreased, but the precise form of this scaling is still unclear, and much more data on large household sizes is required to provide a definitive answer.

#### 5.2.1. Inference on Fully Heterogeneous Populations

Perhaps the holy grail of statistical inference on epidemics is to make use of an individual-level model to describe heterogeneous populations at the limit of granularity. In this respect, Bayesian inference on stochastic mechanistic models using MCMC have perhaps shown the most promise, allowing inference to be made on both transmission parameters and using data augmentation to estimate the infectious period. 

An analysis of the 1861 outbreak of measles in Hagelloch by Neal and Roberts [166] demonstrates the use of a reversible jump MCMC algorithm to infer disease transmission parameters and infectious period, whilst additionally allowing formal comparisons to be made between several nested models. With the uncertainty surrounding model choice, such methodology is vital to enable accurate understanding and prediction. This approach has since been combined with the algorithm of O'Neill and Roberts [165] and used to analyse disease outbreaks such as avian influenza and foot and mouth disease in livestock populations [146, 147, 170] and MRSA outbreaks in hospital wards [171].

Whilst representing the cutting edge of inference on infectious disease processes, these approaches are currently limited by computing power, with their algorithms scaling by the number of infectives multiplied by the number of susceptibles. However, with advances in computer technology expected at an increasing rate, and small approximations made in the calculation of the statistical likelihoods needed in the MCMC algorithms, these techniques may well form the mainstay of epidemic inference in the future.

### 5.3. Inference from Contact Tracing

In livestock diseases, part of the standard response to a case detection is to gather contact tracing information from the farmer. The resulting data are a list of contacts that have been made in and out of the infected farm during a stipulated period prior to the notification of disease [172]. In terms of disease control on a local level, this has the aim of identifying both the source of infection and any presumed susceptibles that might have been infected as a result of the contact. It has been shown that providing the efficiency of following up any contacts to look for signs of disease is high; this is a highly effective method of slowing the spread of an epidemic and finally containing it. 

Much has been written on how contact tracing may be used to decrease the time between infection and detection (notification) during epidemics. However, this focuses on the theoretical aspects of how contact tracing efficiency is related to both epidemic dynamics and population structure (see, e.g., Eames and Keeling [173] Kiss et al. [174], Klinkenberg et al. [175]). In contrast, the use of contact tracing data in inferring epidemic dynamics does not appear to have been well exploited although it was used by the Ministry of Agriculture, Fisheries, and Food (now Defra) to directly infer a spatial risk kernel for foot and mouth disease in 2001. This assumed that the source of infection was correctly identified by the field investigators, thereby giving an empirical estimate of the probability of infection as a function of distance [148, 176, 177]. Strikingly, this shows a high degree of similarity to spatial kernel estimates based on the statistical techniques of Diggle [178] and Kypraios [179] without using contact tracing information. However, Cauchemez et al. [155] make the point that the analysis of imperfect contact tracing data requires more complex statistical approaches, although they abandoned contact tracing information altogether in their analysis of the 2003 SARS epidemic in China. Nevertheless, recent unpublished work has shown promise in assimilating imperfect contact tracing data and case detection times to greatly improve inference, and hence the predictive capability of simulation techniques.

#### 5.3.1. Inference from Distributions over Families of Networks

Qualitative results from simulations indicate that epidemics on networks, for some parameter values, show features that distinguish them from homogeneous models. The principal features are a very variable length slow-growth phase, followed by a rapid increase in the infection rate and a slower decline after the peak [180]. However, in quantitative terms, there is usually very limited information about the underlying network and parameters are often not identifiable. When the details of the network are unknown, but something is known or assumed about its formation, estimation of both the epidemic parameters and parameters for the network itself are in principle possible using MCMC techniques. All the stochastic models for generating networks described in Section 2.7 above realise a distribution over all or some of the 2^*N*(*N*−1)/2^ possible networks. In most cases, this distribution is not tractable; MCMC techniques are in principle still possible but in practice would be too slow without careful design of algorithms.

However, with appropriate assumptions some results can be obtained, which provide some insight into what more could be achieved. When the network is taken to be an Erdös-Rényi graph with unknown parameter *p* and the epidemic is a Markovian SIR, Britton and O'Neill [150] showed that it is possible to estimate the parameters, although they highlight the ever-present challenge of disentangling epidemiological from network parameters. The MCMC algorithm was improved by Neal and Roberts [181] and the extension from SIR to SEIR has been developed by Groendyke et al. [182]. However, the extension to more realistic families of networks remains a challenging problem and will undoubtedly be the subject of exciting future research.

## 6. Discussion

The use of networks is clearly a rapidly growing field in epidemiology. By assessing (and quantifying) the potential transmission routes between individuals in a population, researchers are able to both better understand the observed distribution of infection as well as create better predictive models of future prevalence. We have shown how many of the structural features in commonly used contact networks can be quantified and how there is an increasing understanding of how such features influence the propagation of infection. However, a variety of challenges remain.

### 6.1. Open Questions

Several open problems remain if networks are to continue to influence predictive epidemiology. The majority of these stem from the difficulty in obtaining realistic transmission networks for a range of pathogens. Although some work has been done to elucidate the interconnected structure of sexual encounters (and hence the sexual transmission network), these are still relatively small-scale compared to the population size and suffer from a range of potential biases. Determining comparable networks for airborne infections is a far greater challenge due to the less precise definition of a potential contact.

One practical issue is therefore whether new techniques can be developed that allow contact networks to be assessed remotely. Proximity loggers, such as those used by Hamede and colleagues [12], provide one potential avenue although it would require the technology to become sufficiently robust, portable and cheap that a very large proportion of a population could be convinced to carry one at all times. For many human populations, where the use of mobile phones (which can detect each other via Bluetooth) is sufficiently widespread, there is the potential to use them to gather network information—although the challenges of developing sufficiently generic software should not be underestimated. While these remotely sensed networks would provide unparalleled information that could be obtained with the minimum of effort, there would still be some uncertainty surrounding the nature of each contact.

There is now a growing set of diary-based studies that have attempted to record the personal contacts of a large number of individuals; of these, POLYMOD is currently the most comprehensive [17]. While such egocentric data obviously provides extensive information on individual behaviour, due to the anonymity of such surveys it is not clear how the alters should be connected together. The configuration method of randomly connecting half-links provides one potential solution, but what is ideally required is a more comprehensive method that would allow clustering, spatially localised connections and assortativity between degree distributions to be included and specified.

Associated with the desire to have realistic contact networks for entire populations, comes the need to characterise such networks in a relatively parsimonious manner that provides important insights into the types of epidemiological dynamics that could be realised. Such a characterisation would allow for different networks (from different times or different locations) to be compared in a manner that is epidemiologically significant and would allow artificial networks to be created that matched particular known network features. This clearly relies on both existing measures of network structure (as outlined in Section 3) together with a robust understand of how such features influence the transient epidemic dynamics (as outlined in Section 4.2). However, such a generic understanding of all network features is unlikely to arise for many years. A more immediate challenge is to understand ways in which local network structure (clustering, cliques, and spatially localised connections) influence the epidemiological dynamics.

To date the vast majority of the work into disease transmission on networks has focused on static networks where all links are of equal strength and, therefore, associated with the same basic rate of transmission. However, it is clear that contact networks change over time (both on the short-time scale of who we meet each day, and on the longer time-scale of who our main work and social contacts are), and that links have different weights (such that some contacts are much more likely to lead to the transmission of infection than others). While the simulation of infection on such weighted time-varying networks is feasible, it is unclear how the existing sets of network properties or the existing literature of analytical approaches can be extended to such higher-dimensional networks.

For any methodology to have any substantive use in the field, it is important both to have effective data gathering protocols in place and to have the statistical techniques in place to analyse it. Here, three issues are perhaps most critical. Firstly, data gathering resources are almost always limited. Therefore, carefully designed randomised sampling schemata should be employed to maximise the power of the statistical techniques used to analyse data, rather than having to reply on data augmentation techniques to work around the problems present in ad hoc datasets. This aspect is particularly important when working on network data derived from population samples. Secondly, any inference on both network and infectious disease models should be backed up by a careful analysis of model fit. Although recent advances in statistical epidemiology have given us an unprecedented ability to measure population/disease dynamics based on readily available field data, epidemic model diagnostics are currently in their infancy in comparison to techniques in other areas of statistics. Therefore, it is expected that with the growth in popularity of network models for analysing disease spread, much research effort will be required in designing such methodology.

### 6.2. Conclusions

We have highlighted that the study of contact networks is fundamentally important to epidemiology and provides a wealth of tools for understanding and predicting the spread of a range of pathogens. As we have outlined above, many challenges still exist, but with growing interest in this highly interdisciplinary field and ever increasing sophistication in the mathematical, statistical and remote-sensing tools being used, these problems may soon be overcome. We conclude, therefore, that now is an exciting time for research into network epidemiology as many of the practical difficulties are surmounted and theoretical concepts are translated into results of applied importance in infection control and public health.

## Figures and Tables

**Table 1:** Common notation.

Concept/Measure	Other common names	Our notation	Other common notation
Network	Graph	*G*	
Node	Vertex, point, site, actor	*n*	*v*
Edge	Link, tie, bond	*l*	*e*
Adjacency matrix	Connectivity matrix	*G*_*ij*_	*a*_*ij*_, *A*_*ij*_
Number of nodes	Size of network	*N*	*n*, *S*
Number of edges	Graph size	*L*	*e*, *l*
Centrality		*C*	
Degree	Connectivity	*k*	*d*, *C*_*d*_
Betweenness		*B*_*i*_	bet_*i*_, *C*_*b*_
Degree distribution	Connectivity distribution	*P*(*k*)	*P*_*k*_,*p*_*k*_
Shortest path distance	Geodesic distance	*D*_*i*,*j*_	*d*_*i*,*j*_
Clustering	transitivity	*ϕ*	*c*, Φ
Number of nodes of type A		[*A*]	*n*_*A*_, *N*_*A*_
Number of *A* − *B* pairs		[*AB*]	*n*_*AB*_, *N*_*AB*_
Diameter	Maximal shortest path	Diam(*G*)	max (*D*_*i*,*j*_)
